# Assessment of the Role of Carotid Atherosclerosis in the Association Between Major Cardiovascular Risk Factors and Ischemic Stroke Subtypes

**DOI:** 10.1001/jamanetworkopen.2019.4873

**Published:** 2019-05-31

**Authors:** Sarah Parish, Matthew Arnold, Robert Clarke, Huaidong Du, Eric Wan, Om Kurmi, Yiping Chen, Yu Guo, Zheng Bian, Rory Collins, Liming Li, Zhengming Chen

**Affiliations:** 1 Clinical Trial Service Unit and Epidemiological Studies Unit, Nuffield Department of Population Health, University of Oxford, Oxford, United Kingdom; 2 Medical Research Council Population Health Research Unit, Nuffield Department of Population Health, University of Oxford, Oxford, United Kingdom; 3 Chinese Academy of Medical Sciences, Beijing, China; 4 Department of Epidemiology and Biostatistics, School of Public Health, Peking University Health Science Center, Beijing, China

## Abstract

**Importance:**

A better understanding of the role of atherosclerosis in the development of ischemic stroke subtypes could help to improve strategies for prevention of stroke worldwide.

**Objective:**

To assess the role of carotid atherosclerosis in the association between major cardiovascular risk factors and ischemic stroke subtypes.

**Design, Setting, and Participants:**

The prospective China Kadoorie Biobank cohort study was conducted in the general population of 5 urban and 5 rural areas in China, with a baseline survey obtained between June 2004 and July 2008. A random sample of 23 973 participants with no history of cardiovascular disease at enrollment who had carotid artery ultrasonographic measurements recorded at a resurvey from September 2013 to June 2014 were included. Data analysis was performed from July 1, 2016, to April 10, 2019.

**Exposures:**

Major cardiovascular risk factors (ie, blood pressure [BP], blood lipid levels, smoking, and diabetes).

**Main Outcomes and Measures:**

Carotid ultrasonographic measures of plaque burden (derived from number and maximum size of carotid artery plaques at resurvey) and first ischemic stroke during follow-up (n = 952), with subtyping (data release, August 2018) as lacunar (n = 263), probable large artery (n = 193), probable cardioembolic (n = 66), or unconfirmed (n = 430). Associations between cardiovascular risk factors, carotid plaque burden, and ischemic stroke subtypes were adjusted for age, sex, and geographic area.

**Results:**

The 23 973 participants in the study had a mean (SD) age of 50.6 (10.0) years, and 14 833 (61.9%) were women. Systolic BP had a stronger association (odds ratio [OR] per SD, 1.51; 95% CI, 1.42-1.61) than plaque burden (OR per SD, 1.34; 95% CI, 1.26-1.44) with ischemic stroke, and the associations of systolic BP with each subtype of ischemic stroke were modestly attenuated by adjustment for plaque burden. After adjustment for BP, plaque burden had a stronger association with probable large artery stroke (OR, 1.43; 95% CI, 1.24-1.63) than with lacunar stroke (OR, 1.25; 95% CI, 1.10-1.43) but was not associated with probable cardioembolic stroke (OR, 1.06; 95% CI, 0.83-1.36).

**Conclusions and Relevance:**

Although BP was an important risk factor for all ischemic stroke subtypes, carotid atherosclerosis was an important risk factor only for large artery and lacunar strokes, suggesting that drug treatments targeting atherosclerosis may reduce the risk of stroke subtypes to different extents.

## Key Points

**Question:**

What is the role of carotid atherosclerosis in the association between major cardiovascular risk factors and different ischemic stroke subtypes?

**Findings:**

In this population-based cohort study of Chinese adults with subtyping of incident ischemic strokes, carotid artery ultrasonographic measurements were recorded in 23 973 participants after 8 years’ follow-up. Blood pressure was associated with all ischemic stroke subtypes independently of carotid plaque burden, but after adjustment for blood pressure, carotid plaque burden was associated with probable large artery and lacunar stroke but not with probable cardioembolic stroke.

**Meaning:**

Drug treatments targeting atherosclerosis may affect the risk of ischemic stroke subtypes to different extents.

## Teaser

This population-based cohort study examines the role of carotid atherosclerosis in the association between major cardiovascular risk factors and ischemic stroke.

## Introduction

Hypertension, blood cholesterol levels, cigarette smoking, and diabetes are major risk factors for ischemic heart disease, ischemic stroke, and atherosclerosis.^1,2,3,4,5,6,7^ However, global observational studies have reported that hypertension accounts for a higher population-attributable fraction of ischemic stroke than high blood cholesterol levels, whereas hypertension and high blood cholesterol levels are associated with similar attributable fractions of ischemic heart disease.^8^ The relevance of hypertension and high cholesterol levels for ischemic stroke may vary between stroke subtypes (eg, large artery occlusive stroke, cardioembolic stroke, and lacunar stroke) according to the role of atherosclerosis in the development of the individual ischemic stroke subtype.^9,10^

Atherosclerotic plaques begin as focal thickenings of the intimal layers of the arterial wall that progress with lipid deposition. Damage to the arterial wall leading to hypertrophy, thickening, arterial stiffness, and dysfunction can increase the risk of plaque formation.^1^ Measures of atherosclerotic plaque in the carotid arteries and thickness of the carotid intima-media (cIMT) are readily obtained using carotid ultrasonographic techniques.^1^ Both types of measure improve risk prediction for ischemic heart disease and ischemic stroke independently of major cardiovascular risk factors, but measures of plaque are stronger predictors than cIMT.^1,11,12^ Previous large studies on the role of carotid atherosclerosis in ischemic stroke have typically not collected information on the presence of both carotid artery plaque and cIMT alongside information on major cardiovascular risk factors and ischemic stroke subtypes and so have not investigated the role of atherosclerosis in the association between cardiovascular risk factors and ischemic stroke subtypes.^10,13,14^

The present study of 23 973 Chinese adults investigated (1) the associations of major cardiovascular risk factors with carotid artery plaque and cIMT, (2) the associations of carotid artery plaque and cIMT with subtypes of ischemic stroke, and (3) the role of carotid atherosclerosis as a mediator of the associations between cardiovascular risk factors and the stroke subtypes.

## Methods

The present analyses were conducted from July 1, 2016, to April 10, 2019, and this report follows the Strengthening the Reporting of Observational Studies in Epidemiology (STROBE) guidelines. Ethical approval for the China Kadoorie Biobank study was obtained from the Oxford Tropical Research Ethics Committee (OXTREC) at the University of Oxford and the Chinese Center for Disease Control and Prevention Ethical Review Committee. Ethical approval for the 2013-2014 resurvey were obtained from OXTREC and the Chinese Academy of Medical Sciences/Peking Union Medical College Ethical Review Committee. Approval for both the main study and the resurvey was also granted by the institutional boards at the Chinese Center for Disease Control and Prevention in each of the survey sites. Participants provided written informed consent; they did not receive financial compensation.

### Study Population

Details of the China Kadoorie Biobank design and methods have been previously reported.^15^ Overall, 512 891 adults aged 30 to 79 years were enrolled between June 2004 and July 2008 from 10 diverse areas (5 urban, 5 rural) in China. Information was collected on demographic and socioeconomic status, lifestyle behavior, and medical history using interviewer-administered questionnaires. Physical and biochemical measurements included anthropometry, blood pressure (BP) level, and a random blood glucose level. The interviewer-administered questions on smoking included the frequency, type, and amount of smoking both currently and in the past.

### Resurvey in 2013-2014

In a resurvey of a random sample of surviving participants from September 2013 to June 2014, 25 020 participants completed a follow-up questionnaire and had a second set of physical measurements obtained, together with several clinical measurements not included at baseline. Automated B-mode ultrasonographic screening of the extracranial carotid arteries followed a standard protocol consistent with the Mannheim consensus and yielded a mean cIMT measure of the common carotid artery, the number of carotid plaques (defined as focal thickenings of intima-media >1.5 mm), and the thickness of the largest plaque within 4 segments of the carotid arteries.^16^ The plaque measurements were combined to form a carotid plaque burden, interpretable as an enhanced estimate of the maximum plaque thickness as described in eMethods 1 in the Supplement and a previous report.^16^ A 12-lead electrocardiogram and Mortara digital analysis based on Minnesota definitions provided definite or probable evidence of myocardial infarction and cardiac arrhythmia.^17,18^

At the end of the resurvey, participants were provided a report of their measured values and given an opportunity to discuss their results with a physician. Standard laboratory blood lipid measurements (including directly measured low-density lipoprotein cholesterol [LDL-C] levels) at baseline were available in a subset of 2899 participants. Further details of study measurements are provided in eMethods 1 in the Supplement.

### Disease Outcomes

Incident cases of cardiovascular diseases during follow-up were ascertained through linkage via the unique national identification number to electronic hospital records from the nationwide health insurance system, which had more than 98% coverage across the 10 study areas; established local registries of stroke and coronary heart disease; and local death registries. Active follow-up of any uninsured participants and continued maintenance of linkage ensured that less than 1% of participants were lost to follow-up. Adjudication of strokes and their subtypes was undertaken between January 1, 2014, and August 7, 2018, by abstraction of additional information from medical records and brain imaging reports (available for >92% of strokes with retrieved records), following a defined protocol specifying rules independent of vascular risk factors (eMethods 2 in the Supplement). Diagnosis of stroke was verified using World Health Organization criteria for stroke, defined as rapidly developing clinical signs of focal or global disturbance of cerebral function lasting more than 24 hours or leading to death due to a vascular cause.^19^ Findings in radiologic reports from brain imaging and in medical records were used to classify strokes by their pathologic types. Ischemic strokes were further classified based on the radiologic report into the subtypes lacunar stroke if the report stated that the brain infarct was less than 15 mm in diameter or nonlacunar stroke if the infarct diameter was 15 mm or greater.

In the present report, ischemic stroke was defined as documentation in the electronic health records during follow-up of an ischemic stroke or of a stroke of any type that was confirmed as ischemic during adjudication. Adjudicated, nonlacunar ischemic strokes during follow-up were further subdivided into probable cardioembolic stroke or probable large artery stroke by whether the participant had evidence of cardiac disease by the time of the carotid artery scan, which was specified as hospital admission for ischemic heart disease between baseline and resurvey or evidence of definite or probable myocardial infarction or arrhythmia on the electrocardiogram at resurvey. Ischemic strokes not yet confirmed by the adjudication process were subdivided by whether any relevant records for the participant had yet been retrieved during the process (eMethods 2 in the Supplement).

The study population in this report was restricted to participants with no history of cardiovascular disease at baseline (ie, no reported diagnosis of ischemic heart disease, stroke, or transient ischemic attack) to reduce the possibility that baseline risk factors could have been altered by prevalent cardiovascular disease.^20^ The present analysis included 952 individuals who had a first ischemic stroke and no previous hemorrhagic stroke during the 8 years’ mean follow-up before the carotid artery ultrasonographic examination and the 23 021 participants without a stroke by this time.

### Statistical Analysis

All analyses included basic adjustment for age at carotid artery ultrasonographic examination, sex, and geographic area. Further adjustments to investigate mediation included baseline values of the major cardiovascular risk factors and, in analyses of stroke outcomes, carotid artery measures at resurvey. eMethods 3 in the Supplement provides the correspondence between presented results and a mediation framework.^21^ To evaluate the additional association of diastolic BP (DBP) given systolic BP (SBP), the joint associations of SBP and the residuals of DBP adjusted for SBP (DBP-given-SBP) were considered. For smoking, terms for never, occasional, ex-regular, current regular, and amount smoked currently were included. Data on smoking and BP were available in all participants. Diabetes included self-reported diagnosed diabetes and diabetes detected using random blood glucose measurements at baseline (available for 23 643 participants [98.6%]). Linear regression and Wald *P* values were used to assess the joint associations of cardiovascular risk factors with the carotid measures. For display purposes, the terms for smoking were represented as a combined smoking score.

Logistic regression with likelihood ratio tests and *P* values were used to assess the associations of carotid artery measures with stroke after incremental adjustment for cardiovascular risk factors and to test for trends in odds ratios (ORs) by age and SBP groups. Linear effects and ORs are presented per SD unit of the risk factor or, for binary factors (diabetes and diagnosed hypertension), per SD of the prevalence of the factor. Twice the increase in the log-likelihood on the addition of a term of interest gives a χ^2^_1_ statistic that provides both a significance test for the improvement in fit from including the term and a quantitative measure of the extent to which the added term improves risk prediction. Pearson correlation coefficients are reported. Further details of the statistical methods are provided in eMethods 3 in the Supplement. All analyses used SAS, version 9.4 (SAS Institute Inc). Findings were considered statistically significant at 2-tailed *P* < .05.

## Results

The 23 973 participants included in the present substudy had, at baseline, a mean (SD) age of 50.6 (10.0) years; 14 833 were women (61.9%); and the mean (SD) SBP was 130.6 (20.6) mm Hg (Table). These values were representative of all survivors in the China Kadoorie Biobank study without prior cardiovascular disease by the time of the resurvey (eTable in the Supplement).

**Table.** Characteristics of Participants

Characteristic	No. (%) of Participants		
	Ischemic Stroke During Follow-up		All
	No	Yes	
No.	23 021	952	23 973
Age at baseline, mean (SD), y	50.4 (10.0)	57.4 (9.2)	50.6 (10.0)
Women	14 298 (62.1)	535 (56.2)	14 833 (61.9)
Smoking at baseline, mean (SD), cigarettes/d			
Men	11.4 (12.4)	10.3 (11.1)	11.4 (12.3)
Women	0.2 (1.8)	0.2 (1.4)	0.2 (1.8)
BP at baseline, mean (SD), mm Hg			
Systolic	130.1 (20.3)	143.0 (24.7)	130.6 (20.6)
Diastolic	77.3 (10.9)	81.9 (12.4)	77.5 (11.0)
Prior disease at baseline			
Hypertension diagnosed	1950 (8.5)	224 (23.5)	2174 (9.1)
Diabetes diagnosed	477 (2.1)	58 (6.1)	535 (2.2)
Diabetes diagnosed or detected^a^	949 (4.1)	92 (9.7)	1041 (4.3)
Medication			
Antihypertensive at baseline	671 (2.9)	80 (8.4)	751 (3.1)
At resurvey	1701 (7.4)	223 (23.4)	1924 (8.0)
Lipid-lowering at baseline	27 (0.1)	7 (0.7)	34 (0.1)
At resurvey	137 (0.6)	26 (2.7)	163 (0.7)
Lipid levels at baseline			
No.	2643	256	2899
Cholesterol, mean (SD), mg/dL			
LDL	89.4 (26.1)	97.3 (28.1)	90.0 (26.4)
HDL	48.9 (11.2)	47.6 (11.2)	48.8 (11.2)
Ischemic stroke (nonfatal) during follow-up			
No ischemic stroke	23 021 (100)	0	23 021 (96.0)
Ischemic stroke	NA	952 (100)	952 (4.0)
Nonlacunar stroke	NA	259 (27.2)	259 (1.1)
Lacunar stroke	NA	263 (27.6)	263 (1.1)
Unconfirmed	NA	430 (45.2)	430 (1.8)
Carotid artery measures at resurvey			
cIMT, mean (SD), mm	0.69 (0.16)	0.80 (0.19)	0.70 (0.16)
Carotid plaque burden, mean (SD), mm	0.71 (1.03)	1.50 (1.25)	0.74 (1.05)
No plaque or preplaque	14 395 (62.5)	306 (32.1)	14 701 (61.3)
Preplaque ≥1.0 and <1.5	2061 (9.0)	69 (7.2)	2130 (8.9)
Plaque, mm			
≥1.5 and <3.0	5624 (24.4)	452 (47.5)	6076 (25.3)
≥3.0 and <4.5	856 (3.7)	105 (11.0)	961 (4.0)
≥4.5	85 (0.4)	20 (2.1)	105 (0.4)

Abbreviations: BP, blood pressure; cIMT, carotid intima-media thickness; HDL, high-density lipoprotein; LDL, low-density lipoprotein; NA, not applicable.

SI conversion factor: To convert HDL and LDL cholesterol to millimoles per liter, multiply by 0.0259.

^a^ Detected from baseline random blood glucose level.

Antihypertensive medication was used by 751 participants (3.1%) at baseline and by 1924 individuals (8.0%) at resurvey, and lipid-lowering medication was used by 34 participants (0.1%) at baseline and 163 individuals (0.7%) at resurvey. Mean (SD) cIMT was 0.69 (0.16) mm in participants without a stroke and 0.80 (0.19) mm in those with a stroke (Table). Carotid artery plaque (>1.5 mm) was present in 6565 of 23 021 (28.5%) participants without a stroke and in 577 of 952 (60.6%) individuals with a stroke. A total of 54.8% of the 952 ischemic stroke cases were adjudicated as either lacunar (263 cases [27.6%]) or nonlacunar (259 cases [27.2%]); 430 cases (45.2%) remained unconfirmed.

### Risk Factors and Carotid Measures

**Figure 1.**
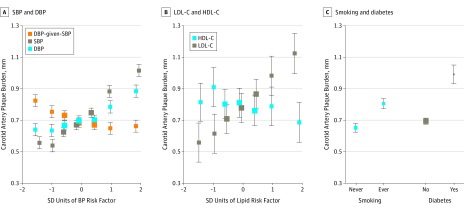
Associations of Major Cardiovascular Risk Factors With Carotid Artery Plaque Burden in Participants Without Stroke at Resurvey Adjusted mean carotid artery plaque burden level by blood pressure (BP) (A), cholesterol levels (B), and smoking and diabetes (C). Blood pressure, smoking, and diabetes associations were determined in 23 021 participants; cholesterol level associations were determined in 2643 participants with measurements at baseline. Associations were adjusted for age, sex, and geographic area. DBP indicates diastolic blood pressure; HDL-C, high-density lipoprotein cholesterol; LDL-C, low-density lipoprotein cholesterol; and SBP, systolic blood pressure. SDs are SBP, 21 mm Hg; DBP, 11 mm Hg; the residuals of DBP adjusted for SBP (DBP-given-SBP), 7 mm Hg; LDL-C, 26 mg/dL; and HDL-C, 11 mg/dL (to convert HDL-C and LDL-C to millimoles per liter, multiply by 0.0259). Error bars indicate 95% CI.

**Figure 2.**
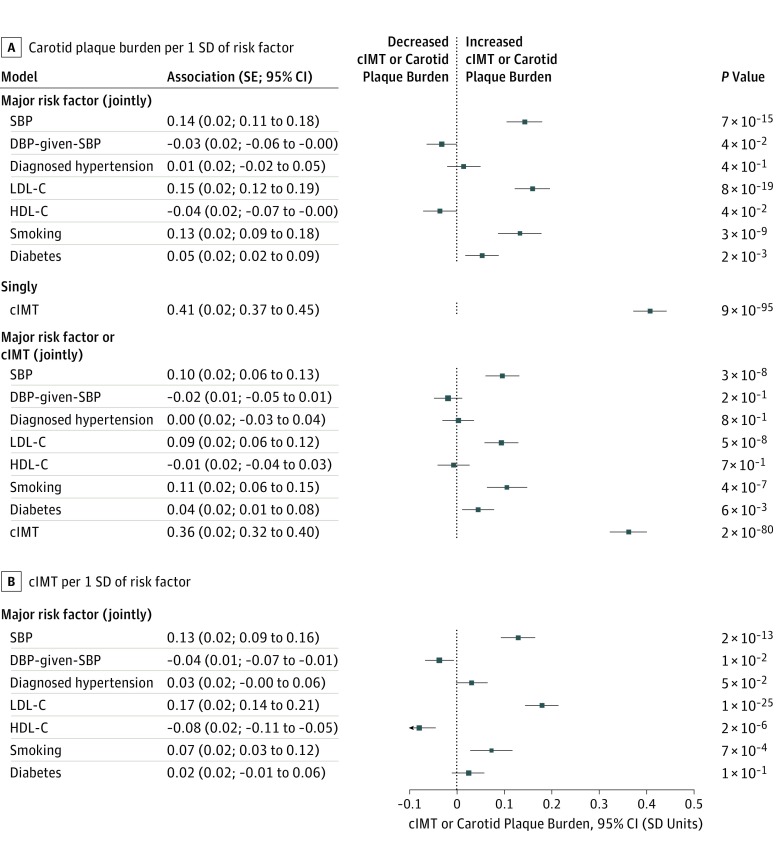
Joint Associations of Major Risk Factors Measured at Baseline With Carotid Plaque Burden and Carotid Intima-Media Thickness (cIMT) in 2643 Participants Without Cardiovascular Disease at Resurvey Associations with carotid plaque burden (A) and cIMT (B) were adjusted for age, sex, and geographic area. The smoking score (scaled to have an SD of 1) includes the terms for smoking status: never, occasional, ex-regular, or current regular smoker with the number of cigarettes smoked currently. For example, in the major risk factors joint model for carotid artery plaque burden (A), the smoking score is 0 for never smokers, −0.16 for occasional smokers, 0.49 for ex-smokers, and 1.44 plus 0.04 per cigarette per day for current smokers. Thus, a current smoker of 15 cigarettes per day would have a score of approximately 2. For consistency with other factors, the diabetes and diagnosed hypertension associations are displayed as the association per SD of the condition prevalence; to convert to the effect with having the condition, divide the values in the figure by the SD of the respective prevalence. SDs are diagnosed hypertension prevalence, 0.29, diabetes prevalence, 0.20, carotid plaque burden, 1.1 mm, and cIMT, 0.16 mm; SBP, 21 mm Hg; the residuals of DBP adjusted for SBP (DBP-given-SBP), 7 mm Hg; LDL-C, 26 mg/dL; and HDL-C, 11 mg/dL (to convert HDL-C and LDL-C to millimoles per liter, multiply by 0.0259). DBP indicates diastolic blood pressure; HDL-C, high-density lipoprotein cholesterol; LDL-C, low-density lipoprotein cholesterol; and SBP, systolic blood pressure.

Among the 23 021 participants without a stroke, the associations of baseline BP and blood lipid levels with both of the carotid measures were broadly linear. Likewise, smoking and diabetes were associated with higher levels of both carotid measures (Figure 1; eFigure 1 in the Supplement). In analyses of the joint associations of the major cardiovascular risk factors at baseline with the carotid measures in the subset with lipid measurements, the strongest risk factors for plaque burden were LDL-C (0.15; SE, 0.02; *P* = 8 × 10^−19^) and SBP (0.14; SE, 0.02; *P* = 7 × 10^−15^) SD plaque burden per SD of the risk factor. Smoking was also associated with plaque burden (0.13; SE, 0.02; *P* = 3 × 10^−9^) (Figure 2A).

The pattern of association of most risk factors with cIMT (Figure 2B) was similar to that with plaque burden. However, the high-density lipoprotein cholesterol level was inversely associated with cIMT (−0.08; SE, 0.02; *P* = 2 × 10^−6^) but had only a borderline statistically significant association with plaque burden (−0.04; SE, 0.02; *P* = .04). The cIMT was associated with plaque burden (0.41; SE, 0.02; *P* = 9 × 10^−95^) and, when included as a risk factor in a joint model, the associations of other cardiovascular risk factors with plaque burden were reduced by 20% to 40% (Figure 2).

### Carotid Measures and Ischemic Stroke

**Figure 3.**
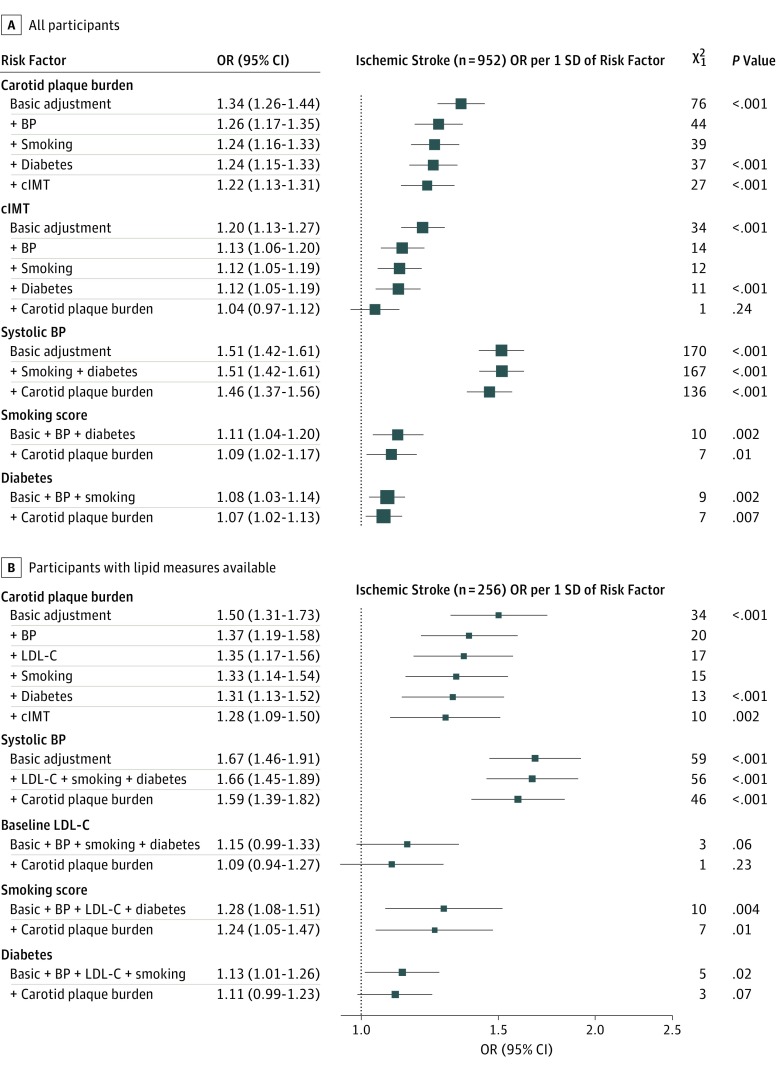
Associations of Carotid Measures and Major Cardiovascular Risk Factors With Ischemic Stroke Associations were determined for all participants (n = 23 973) (A) and those with lipid levels available (n = 2899) (B). The size of each square is proportional to the amount of statistical information. Basic adjustment was age, sex, and geographic area. *P* values are provided only for key comparisons to aid focus. Smoking adjustment is for terms in the smoking score. For consistency with other factors, diabetes odds ratios (ORs) displayed are per SD of diabetes prevalence; ORs associated with having diabetes are exp(log[displayed OR]/0.20). BP indicates blood pressure; cIMT, carotid intima-media thickness; and LDL-C, low-density lipoprotein cholesterol.

Both plaque burden and cIMT values showed approximately log-linear positive associations with risk of ischemic stroke after adjustment for age, sex, and geographic area (eFigure 2 in the Supplement). Overall, a 1-SD higher plaque burden was associated with an adjusted OR of 1.34 (95% CI, 1.26-1.44; χ^2^_1_ = 76; *P* < .001) for ischemic stroke (Figure 3). Additional adjustment for BP, smoking, and diabetes reduced the OR to 1.24 (95% CI, 1.15-1.33) and reduced the χ^2^ value from 76 to 37, but the association remained statistically significant (OR, 1.22; 95% CI, 1.13-1.31; χ^2^_1_ = 27; *P* < .001) even after further adjustment for cIMT.

The cIMT had a Pearson correlation coefficient of only 0.3 with plaque burden and had a weaker association than plaque burden with ischemic stroke (OR per 1 SD–greater cIMT: 1.20; 95% CI, 1.13-1.27; χ^2^_1_ = 34; *P* < .001) (Figure 3) and was no longer independently associated with ischemic stroke after adjustment for the cardiovascular risk factors and plaque burden (OR, 1.04; 95% CI, 0.97-1.12; χ^2^_1_ = 1; *P* = .24). There was a significant trend toward higher ORs for ischemic stroke per 1 SD–higher carotid measures at younger ages and lower SBP levels (eg, the OR for ischemic stroke with plaque burden varied from 1.23; 95% CI, 1.11-1.37 at ages 70-89 years to 1.52; 95% CI, 1.32-1.76 at ages 40-39 years, *P* = .02 for trend, and from 1.20; 95% CI, 1.10-1.31 at SBP ≥140 mm Hg to 1.44; 95% CI, 1.23-1.69 at SBP <120 mm Hg, *P* = .02 for trend) (eFigure 3 in the Supplement).

### Risk Factors and Ischemic Stroke

Baseline SBP level had a substantially stronger association (OR per SD, 1.51; 95% CI, 1.42-1.61; χ^2^_1_ = 170; *P* < .001) than plaque burden (OR per SD, 1.34; 95% CI, 1.26-1.44; χ^2^_1_ = 76; *P* < .001) with ischemic stroke, and the association was only slightly attenuated to 1.46 (95% CI, 1.37-1.56; χ^2^_1_ = 136; *P* < .001) by adjustment for other cardiovascular risk factors and plaque burden (Figure 3). The smoking score was more weakly associated (OR per SD, 1.11; 95% CI, 1.04-1.20; χ^2^_1_ = 10; *P* = .002) than plaque burden with ischemic stroke, as was diabetes (OR per SD, 1.08; 95% CI, 1.03-1.14; χ^2^_1_ = 9; *P* = .002). The associations of ischemic stroke with smoking score and diabetes were also only slightly attenuated by adjustment for plaque burden. Limited data were available on baseline LDL-C level, but its association with ischemic stroke (OR per SD, 1.15; 95% CI, 0.99-1.33; χ^2^_1_ = 3; *P* = .06) was considerably weaker than the associations of SBP and plaque burden (Figure 3B).

### Carotid Measures and Ischemic Stroke Subtypes

**Figure 4.**
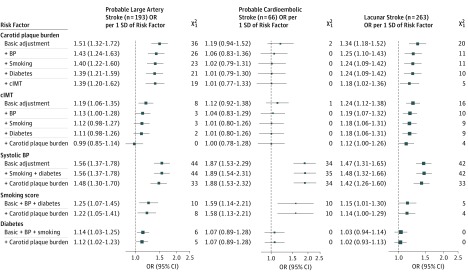
Associations of Carotid Measures and Major Cardiovascular Risk Factors With Ischemic Stroke Subtypes The χ^2^_1_ values 11 and higher are *P* < .001. Basic adjustment was age, sex, and geographic area. Smoking adjustment is for terms in the smoking score. For consistency with other factors, diabetes odds ratios (ORs) displayed are per SD of diabetes prevalence; ORs associated with having diabetes are exp(log[displayed OR]/0.20). BP indicates blood pressure; cIMT, carotid intima-media thickness; and LDL-C, low-density lipoprotein cholesterol.

The association of plaque burden with ischemic stroke subtypes was strongest for probable large artery stroke (n = 193; OR per SD, 1.51; 95% CI, 1.32-1.72; χ^2^_1_ = 36) and weaker for lacunar stroke (n = 263; OR per SD, 1.34; 95% CI, 1.18-1.52; χ^2^_1_ = 20) and probable cardioembolic stroke (n = 66; OR per SD, 1.19; 95% CI, 0.94-1.52; χ^2^_1_ = 2) (Figure 4). After adjustment for BP, associations of plaque burden persisted with large artery stroke (OR per SD, 1.43; 95% CI, 1.24-1.63; χ^2^_1_ = 26) and lacunar stroke (OR per SD, 1.25; 95% CI, 1.10-1.43; χ^2^_1_ = 11) but not for probable cardioembolic stroke (OR per SD, 1.06; 95% CI, 0.83-1.36; χ^2^_1_ = 0). Adjustment for all of the major cardiovascular risk factors accounted for about half of the strength of the associations of plaque burden with probable large artery stroke and lacunar stroke (χ^2^_1_ statistics decreasing from 36 to 21 and from 20 to 10, respectively). The cIMT was weakly associated with both of these stroke subtypes (OR per SD, 1.19; 95% CI, 1.06-1.35; χ^2^_1_ = 8 for large artery stroke and 1.24; 95% CI, 1.12-1.38; χ^2^_1_ = 16 for lacunar stroke), but neither plaque burden nor cIMT was associated with probable cardioembolic stroke after adjustment for BP.

Systolic BP and smoking score were each associated with all ischemic stroke subtypes but had stronger ORs with probable cardioembolic stroke (OR per SD, 1.87; 95% CI, 1.53-2.29, χ^2^_1_ = 34 for SBP, and OR per SD, 1.59; 95% CI, 1.14-2.21; χ^2^_1_ = 10 for smoking score) than with other subtypes. Diabetes had a weak association with probable large artery stroke only (OR per SD, 1.14; 95% CI, 1.03-1.25; χ^2^_1_ = 6). The LDL-C level was not available for a sufficient number of participants to be considered in this analysis of ischemic stroke subtypes.

The associations of plaque burden and cardiovascular risk factors with the 430 ischemic strokes that remained unconfirmed (243 because records had not been retrieved, 187 in which the retrieved records lacked evidence) were similar to the associations with all ischemic strokes (eFigure 4 in the Supplement; Figure 3). When the presence of carotid plaque was considered instead of plaque burden, similar patterns of association were observed, both of risk factors with plaque (LDL-C and SBP remained the strongest factors; eFigure 5 in the Supplement) and of plaque with strokes (eFigure 6 in the Supplement), but the association statistics were weaker for the presence of plaque. For example, the χ^2^_1_ value for the association presence of plaque with ischemic stroke was 57 (eFigure 6 in the Supplement), whereas the χ^2^_1_ value for the association of plaque burden with ischemic stroke was 76 (Figure 3A).

## Discussion

This large study has identified different patterns of association of carotid artery atherosclerosis and BP with probable ischemic stroke subtypes. Low-density lipoprotein cholesterol and BP levels measured a mean of 8 years before carotid ultrasonographic examination demonstrated similar strengths of association with plaque burden. However, LDL-C was a weaker risk factor than plaque burden for all ischemic strokes, whereas BP was a stronger risk factor than plaque burden (Figure 3). Blood pressure was associated with all ischemic stroke subtypes before and after adjustment for carotid artery measures and other major cardiovascular risk factors, but its association was strongest with probable cardioembolic stroke (Figure 4). Plaque burden showed a strength of association similar to that of BP for probable large artery stroke, and this association was only partially attenuated by adjustment for BP. By contrast, plaque burden was not associated with probable cardioembolic stroke after adjustment for BP.

The reductions of less than 20% in the χ^2^ for the strength of association of BP with each subtype of ischemic stroke on adjustment for plaque burden provide an indication of the limited extent to which the associations of BP with ischemic stroke subtypes were mediated through atherosclerosis. Carotid measurements performed on different occasions (not currently available) might remove some measurement error and account for a somewhat larger proportion of the associations of BP, but the small percentages accounted for by measurements from a single carotid artery scan suggest that BP has a substantial association with all types of stroke independent of its association with plaque burden. Smoking and diabetes were also associated with plaque burden, consistent with results in previous studies.^3,4,7^

Adjustment for measured values of BP and other cardiovascular risk factors accounted for about half of the χ^2^ values for both the strong association of plaque burden with probable large artery stroke and the weaker association of plaque burden with lacunar stroke (Figure 4); adjustment for lifelong levels of those risk factors would account for an even higher proportion of the associations.^22^ Hence, these cardiovascular risk factors may account for most of the associations of plaque burden with these ischemic stroke subtypes. Therefore, control of such risk factors, which has been shown to delay the progression of cIMT and plaque,^6,23,24,25,26,27^ should be the main strategy to reduce carotid atherosclerosis.

Plaque burden was a risk factor for ischemic stroke after adjustment for the cardiovascular risk factors, showing that, when data are available, plaque burden should be regarded as an additional risk factor when evaluating risk and the need for drug treatment to reduce the risk of cardiovascular disease.^28^ The observational associations in the present study support BP control as the most important factor for the prevention of stroke because BP was associated with ischemic stroke risk independently of plaque burden, as well as being an important risk factor for plaque burden. However, the findings suggest that other drug treatments targeting reduction in atherosclerosis, such as lipid-lowering and antiplatelet therapy, may have less benefit for the prevention of cardioembolic strokes than for the prevention of large artery strokes.

The present findings in a Chinese population are well supported by a Canadian registry study reporting that, from 2002 to 2012, with the increasingly intensive management of atherosclerotic risk factors (eg, LDL-C lowering with statin therapy and preventive revascularization), the incidence of large artery and small vessel stroke had declined while the incidence of cardioembolic stroke had risen.^14^ Complementing these observational associations, in findings from genetics consortia, genetically elevated LDL-C levels were associated with a higher risk of large artery stroke but not consistently with a risk of small artery stroke or cardioembolic stroke; however, more data are needed to reliably confirm these distinctions.^29,30^

A study using magnetic resonance imaging–verified lacunar infarcts found that all of the major atherosclerotic risk factors were associated with a higher risk of lacunar stroke overall. However, the study also highlighted that lacunar or small artery strokes are often misdiagnosed and include distinct subtypes that may have different risk factors.^31^ The finding in the present study of a stronger association of plaque burden with probable large artery stroke than with lacunar stroke is also consistent with findings from a previous study of approximately 3000 incident ischemic strokes within 5 stroke registers in the United Kingdom, Sweden, and Australia.^10^ The latter study suggested that nonatherosclerotic vascular disease may be the major cause of lacunar stroke.

The cIMT had a Pearson correlation coefficient of only 0.3 with plaque burden and had a weaker association than plaque burden with overall and nonlacunar ischemic stroke but had a similar weak strength of association with lacunar stroke. However, high-density lipoprotein cholesterol was associated with cIMT (*P* = 2 × 10^−6^) but not with plaque burden, consistent with a previous report.^32^ High-density lipoprotein cholesterol may be more closely associated with vascular mechanisms linked to cIMT than with atherosclerosis. Other large studies of carotid artery imaging and ischemic stroke have not clarified the relative importance of carotid artery plaque vs cIMT for risk of ischemic stroke subtypes. In the Atherosclerosis Risk in Communities study, which involved 13 000 individuals with carotid artery measures who were followed up serially for incident cardiovascular disease since 1987, cIMT showed a slightly stronger association with nonlacunar strokes (n = 358) than with lacunar strokes (n = 131), but associations of carotid artery plaque with stroke subtypes were not assessed.

### Limitations

The study has limitations. Brain imaging data were available for more than 92% of adjudicated strokes and provided reliable distinction between lacunar and nonlacunar strokes. However, the adjudication process did not distinguish large artery and cardioembolic nonlacunar strokes; therefore, nonlacunar strokes could be categorized only into probable large artery and probable cardioembolic using observational evidence of cardiac disease. In China, cardioembolic stroke is relatively uncommon and cardiovascular risk factors are managed less intensively.^33^ The present study was also limited to investigating the associations of carotid artery measures with the prevalence of nonfatal stroke during the 8 years before the carotid artery measurement and so may have been subject to reverse causality bias in carotid artery measurements through greater use of drug treatment following a stroke. However, an advantage in the present study in Chinese adults was the relatively low use of BP- or lipid-lowering medication,^34^ as found in another Chinese study,^35^ and hence the influence of any such bias is likely to be small.

## Conclusions

The present study including measurements of plaque burden in 23 973 individuals and data on stroke subtypes suggests that carotid artery atherosclerosis is an important risk factor chiefly for large artery and lacunar stroke. By contrast, BP is an important risk factor both for atherosclerosis and for all ischemic stroke subtypes. Carotid artery imaging in large-scale studies is feasible and informative for distinguishing the underlying pathophysiologic characteristics of ischemic stroke subtypes, and data from such imaging may lead to better understanding of the potential benefits of different drug treatments for prevention of different subtypes of ischemic stroke.

## Notes

Supplement. **eMethods 1.** Further Details of Study Measurements **eMethods 2.** Adjudication and Subtyping of Incident Stroke Events **eMethods 3.** Further Statistical Methods **eReferences** **eTable** Characteristics of Participants in the Carotid Artery Imaging Study compared with in the Whole China Kadoorie Biobank Study **eFigure 1.** Associations of Major Cardiovascular Risk Factors at Baseline with Carotid Measures, in Participants Without Cardiovascular Disease by Resurvey **eFigure 2.** Associations of Carotid Plaque Burden and Carotid Intima-media Thickness with Ischemic Stroke **eFigure 3.** Associations of Carotid Plaque Burden and Carotid Intima-media Thickness With Ischemic Stroke, by Groups of Age at Resurvey and Baseline Systolic Blood Pressure (SBP) **eFigure 4.** Associations of Carotid Measures and Major Cardiovascular Risk Factors With Unconfirmed Ischemic Stroke in 23 973 Participants **eFigure 5.** The Joint Associations of Major Cardiovascular Risk Factors Measured at Baseline With the Presence Of Carotid Plaque (>1.5 mm) in 2643 Participants Without Cardiovascular Disease by Resurvey **eFigure 6.** Association of Presence of Carotid Plaque (>1.5 mm) With Ischemic Stroke: A in All 23 973 Participants, B in 2899 Participants With Lipid Measurements
