# Supplementary material for: Assessment of the Role of Carotid Atherosclerosis in the Association Between Major Cardiovascular Risk Factors and Ischemic Stroke Subtypes
Source: JAMA Netw Open. 2019 May 31;2(5):e194873. doi: 10.1001/jamanetworkopen.2019.4873 (PMC6547114; doi:10.1001/jamanetworkopen.2019.4873)
Supplement: Supplement. — eMethods 1. Further Details of Study Measurements eMethods 2. Adjudication and Subtyping of Incident Stroke Events eMethods 3. Further Statistical Methods eReferences eTable Characteristics of Participants in the Carotid Artery Imaging Study compared with in the Whole China Kadoorie Biobank Study eFigure 1. Associations of Major Cardiovascular Risk Factors at Baseline with Carotid Measures, in Participants Without Cardiovascular Disease by Resurvey eFigure 2. Associations of Carotid Plaque Burden and Carotid Intima-media Thickness with Ischemic Stroke eFigure 3. Associations of Carotid Plaque Burden and Carotid Intima-media Thickness With Ischemic Stroke, by Groups of Age at Resurvey and Baseline Systolic Blood Pressure (SBP) eFigure 4. Associations of Carotid Measures and Major Cardiovascular Risk Factors With Unconfirmed Ischemic Stroke in 23 973 Participants eFigure 5. The Joint Associations of Major Cardiovascular Risk Factors Measured at Baseline With the Presence Of Carotid Plaque (>1.5 mm) in 2643 Participants Without Cardiovascular Disease by Resurvey eFigure 6. Association of Presence of Carotid Plaque (>1.5 mm) With Ischemic Stroke: A in All 23 973 Participants, B in 2899 Participants With Lipid Measurements [file jamanetwopen-2-e194873-s001.pdf]

## Supplementary Online Content

Parish S, Arnold M, Clarke R, et al; China Kadoorie Biobank Collaborative Group. Assessment of the role of carotid atherosclerosis in the association between major cardiovascular risk factors and ischemic stroke subtypes. *JAMA Netw Open*. 2019;2(5): e194873. doi:10.1001/jamanetworkopen.2019.4873

**eMethods 1.** Further Details of Study Measurements

**eMethods 2.** Adjudication and Subtyping of Incident Stroke Events

**eMethods 3.** Further Statistical Methods

**eReferences**

**eTable.** Characteristics of Participants in the Carotid Artery Imaging Study compared with in the Whole China Kadoorie Biobank Study

**eFigure 1.** Associations of Major Cardiovascular Risk Factors at Baseline with Carotid Measures, in Participants Without Cardiovascular Disease by Resurvey

**eFigure 2.** Associations of Carotid Plaque Burden and Carotid Intima-media Thickness with Ischemic Stroke

**eFigure 3.** Associations of Carotid Plaque Burden and Carotid Intima-media Thickness With Ischemic Stroke, by Groups of Age at Resurvey and Baseline Systolic Blood Pressure (SBP)

**eFigure 4.** Associations of Carotid Measures and Major Cardiovascular Risk Factors With Unconfirmed Ischemic Stroke in 23 973 Participants

**eFigure 5.** The Joint Associations of Major Cardiovascular Risk Factors Measured at Baseline With the Presence Of Carotid Plaque (>1.5 mm) in 2643 Participants Without Cardiovascular Disease by Resurvey

**eFigure 6.** Association of Presence of Carotid Plaque (>1.5 mm) With Ischemic Stroke: A in All 23 973 Participants, B in 2899 Participants With Lipid Measurements

This supplementary material has been provided by the authors to give readers additional information about their work.

© 2019 Parish S et al. *JAMA Network Open.*

## eMethods 1: Further details of study measurements

### Carotid artery measurements

Automated B-mode ultrasound screening of the extra-cranial carotid arteries (using a Panasonic Cardio-Health Station implementing edge-detection software) was undertaken following a standard protocol consistent with the Mannheim consensus, and involved scanning both carotid arteries with automated measurements of carotid intima-media thickness (cIMT) and semi-automated recording of plaques.[1] cIMT was measured in the distal 1cm of the common carotid artery (CCA) just before the bifurcation at four predefined angles (two on each side) based on the Meijer Carotid Arc, using an in-built electronic transducer position guidance, including the right CCA at 150º and 120º and the left CCA at 210º and 240º. Hence, mean cIMT was estimated as the mean of four measurements per person. The number of carotid plaques (defined as focal thickenings of intima-media >1.5 mm) and the thickness of the largest plaque within four segments of the carotid arteries were recorded.[1] Carotid plaque burden was derived by first standardising the plaque number and maximum thickness (i.e., dividing each by its standard deviation [SD]) and estimating the average, then multiplying the average value by the SD of the maximum plaque thickness to provide a plaque burden recorded in millimetre units (i.e. interpretable as an enhanced estimate of the maximum plaque thickness).

### Other measurements

Blood pressure was measured twice using a Omron UA-779 digital sphygmomanometer after participants had remained at rest in a seated position for at least 5 minutes. If the difference between the two measurements was more than 10 mmHg for SBP, a third measurement was made and the last two measurements were recorded. The mean of the two recorded values was used for analysis.

Except in one study area, where the protocol specified fasting by all participants, initial screening for hyperglycaemia involved immediate on-site testing of non-fasting blood glucose using the SureStep Plus meter (LifeScan, Milpitas, CA, USA). Participants with nonfasting glucose levels ≥7.8 and <11.1 mmol/l were invited to return for a fasting blood glucose test the next day. Screen-detected diabetes was defined as no prior history of diabetes and any of: (1) a random blood glucose level ≥7.0 mmol/l and a fasting time >8 h; (2) a random blood glucose level ≥11.1mmol/l and a fasting time <8 h; or (3) a fasting blood glucose level ≥7.0 mmol/l.

© 2019 Parish S et al. *JAMA Network Open.*

Low and high density lipoprotein cholesterol measurements in baseline samples were available for a subset of participants overlapping a nested case-control study of the association of genome-wide panel and biochemistry markers with cardiovascular disease. Data were available for 2899 participants in the present study (256 with an ischemic stroke and 2643 without).

© 2019 Parish S et al. *JAMA Network Open.*

## eMethods 2: Adjudication and subtyping of incident stroke events

Incident cases of stroke were identified using the linked electronic health records. To confirm the accuracy of pathological stroke types and to classify strokes into subtypes, medical records of all reported stroke cases were sought for independent verification and diagnosis by a panel of trained neurologists in China.

All hospital admissions with reported stroke episodes to be verified were collated in a central data repository in the co-ordinating centre at Oxford, UK prior to loading onto a portable computer tablet ("Portable Verification Device") in relevant batches for each participating hospital. Local research assistants visited the hospitals where stroke events had been reported to retrieve associated medical records. Once retrieved and matched with the reported event, the relevant sections of the medical records were photographed using the Portable Verification Device. The information collected included photographs of the admission notes, summary sheets, reports of key diagnostic tests (including brain CT/MRI results) recorded during their hospital stay. Brain imaging reports were available for over 92% of retrieved stroke events. For each reported stroke episode the dates of admission and discharge were checked and relevant clinical data verified and recorded electronically using a standardised data entry form. The data collected included primary and secondary diagnoses of stroke at discharge (including stroke pathological types if available), vital status, other events occurring during hospitalisation, diagnostic tests and their results, and all prescribed medication recorded in the notes. The details of the relevant medical records were uploaded onto a secure internet website for subsequent adjudication.

Trained neurologists in China used a secure internet-based Case Adjudication System for clinical Events (i-CASE) system to adjudicate all verified stroke diagnoses using the World Health Organization criteria for stroke (defined as "rapidly developing clinical signs of focal or global disturbance of cerebral function, lasting more than 24 hours or leading to death due a vascular cause"). Adjudicators used findings in radiological reports on brain imaging (including the presence of hemorrhage or ischemia, laterality of lesions, and location in the brain) and other relevant findings in the patient's medical records to classify strokes into the pathological types: ischemic stroke,

© 2019 Parish S et al. *JAMA Network Open.*

intracranial hemorrhage or subarachnoid hemorrhage. Confirmed ischemic strokes were further classified into the subtypes: lacunar stroke if the radiological report stated an infarct < 15 mm in diameter diagnosed as a lacunar infarct;[2] or non-lacunar stroke if not. All verified stroke cases that remained unconfirmed after adjudication were referred for final review by both Chinese- and English-speaking clinicians in the study coordinating centre in Oxford.

The most recent data release available (Release 15, August 2018) provides reliable confirmation and subtyping of a high proportion of strokes but has not yet consolidated information to reliably refute that a participant had a stroke, as this requires the further complex process of ascertainment that all potential strokes for a participant have been adjudicated. (Medical records for patients in China are not centrally consolidated and may be spread across several hospitals.)

© 2019 Parish S et al. *JAMA Network Open.*

## eMethods 3: Further statistical methods

### Further details on adjustments

Analyses of associations between carotid measures and cardiovascular risk factors included basic adjustment of age-at-resurvey (5 year groups from <45 to ≥ 80, as a categorical variable) x sex x region. Analyses of associations between carotid measures and cardiovascular events included basic adjustment of age-at-resurvey (<5 year groups from <45 to ≥ 80, as a categorical variable) x sex + region. (The slightly more limited adjustment for the interactions with age in the outcome analyses was chosen to avoid over-adjustment in analyses with more limited power.) Additional adjustment for baseline blood pressure included systolic blood pressure (SBP), SBP x age-at-resurvey (<60, 60-69, ≥70 years) diastolic blood pressure (DBP), DBP x age-at-resurvey (<60, 60-69, ≥70 years) and diagnosed-hypertension at baseline.

### Grouping of measures

For examination of the shape of the relationship between cardiovascular risk factors and the carotid measures, continuous valued risk factors were divided into 7 groups defined by the 10$^{th}$, 20$^{th}$, 40$^{th}$, 60$^{th}$, 80$^{th}$ and 90$^{th}$ percentiles of their respective distributions. For examination of the shape of the relationship between the carotid measures and stroke risk, participants were ranked by the respective carotid measure and divided into 6 groups so that an approximately equal number (~158) of strokes was included in each group.

### Correspondence of presented results to a mediation framework

Following the casual steps approach summarised by McKinnon et al.[3], 4 are steps are required to assess mediation between an independent variable and a dependent variable, i.e., in the present context between cardiovascular risk factors and stroke by plaque burden. The correspondence between the presented analyses and the 4 steps is indicated below. In interpretting these regression associations, based on measurements of risk factors and carotid parameters at a single time, consideration needs to be given to the impact of measurement error and short-term variability, as has been done in the Discussion.

*1. "A significant relation of the independent variable to the dependent variable".*[3]

© 2019 Parish S et al. *JAMA Network Open.*

Figures 3 and 4, rows showing the associations of cardiovascular risk factors with strokes, without adjustment for plaque burden.

*2. "A significant relation of the independent variable to the hypothesized mediating variable".*[3]

Figure 1 showing the association of cardiovascular risk factors with plaque burden.

*3. "The mediating variable must be significantly related to the dependent variable when both the independent variable and mediating variable are predictors of the dependent variable".*[3]

Plaque burden must be significantly related to stroke when cardiovascular risk factors are also in the model, which is shown in Figures 3 and 4, in the rows for the association of plaque burden with strokes after adjustment for cardiovascular risk factors.

*4. "The coefficient relating the independent variable to the dependent variable must be larger (in absolute value) than the coefficient relating the independent variable to the dependent variable in the regression model with both the independent variable and the mediating variable predicting the dependent variable".*[3]

In Figures 3 and 4 the odds ratios for the association of cardiovascular risk factors with strokes are larger when plaque burden is not adjusted for, than when it is adjusted for.

## eReferences

1. Clarke R, Du H, Kurmi O, Parish S, Yang M, Arnold M, et al. Burden of carotid artery atherosclerosis in Chinese adults: Implications for future risk of cardiovascular diseases. Eur J Prev Cardiol. 2017; 24:647-56.
2. Norrving B. Long-term prognosis after lacunar infarction. Lancet Neurology 2003; 2: 238–45
3. MacKinnon DP, Fairchild AJ, Fritz MS. Mediation analysis. Annual Review of Psychology. 2007; 58: 593-614.

© 2019 Parish S et al. *JAMA Network Open.*

| Characteristic | All survivors at mid-point of resurvey (1 Jan 2014) | Carotid artery imaging study |
|---|---|---|
| Participants | 485204 | 25020 |
| Participants with prior cardiovascular disease | 21875 | 1047 |
| ***Among participants without prior cardiovascular disease*** | | |
| Participants | 463329 | 23973 |
| Age at baseline, years | 50.5 (10.3) | 50.6 (10.0) |
| Female | 278280 (60.1) | 14833 (61.9) |
| **Smoking at baseline** | | |
| Men: Cigarettes per day | 12.1 (12.0) | 11.4 (12.3) |
| Women: Cigarettes per day | 0.2 (1.8) | 0.2 (1.8) |
| **Blood pressure at baseline** | | |
| Systolic BP, mmHg | 129.9 (20.5) | 130.6 (20.6) |
| Diastolic BP, mmHg | 77.5 (10.9) | 77.5 (11.0) |
| **Prior disease at baseline** | | |
| Hypertension diagnosed | 43568 (9.4) | 2174 (9.1) |
| Diabetes diagnosed | 11557 (2.5) | 535 (2.2) |
| Diabetes diagnosed or detected* | 23360 (5.0) | 1041 (4.3) |
| **Ischemic stroke (non-fatal) during follow-up** | | |
| Ischemic stroke | 19595 (4.2) | 952 (4.0) |
| Values are means (SD) or N (%). BP=blood pressure. SD=standard deviation. *Detected from baseline random blood glucose level. | | |
| **eTable: Characteristics of Participants in the Carotid Artery Imaging Study Compared to the Whole China Kadoorie Biobank Study** | | |

© 2019 Parish S et al. *JAMA Network Open.*

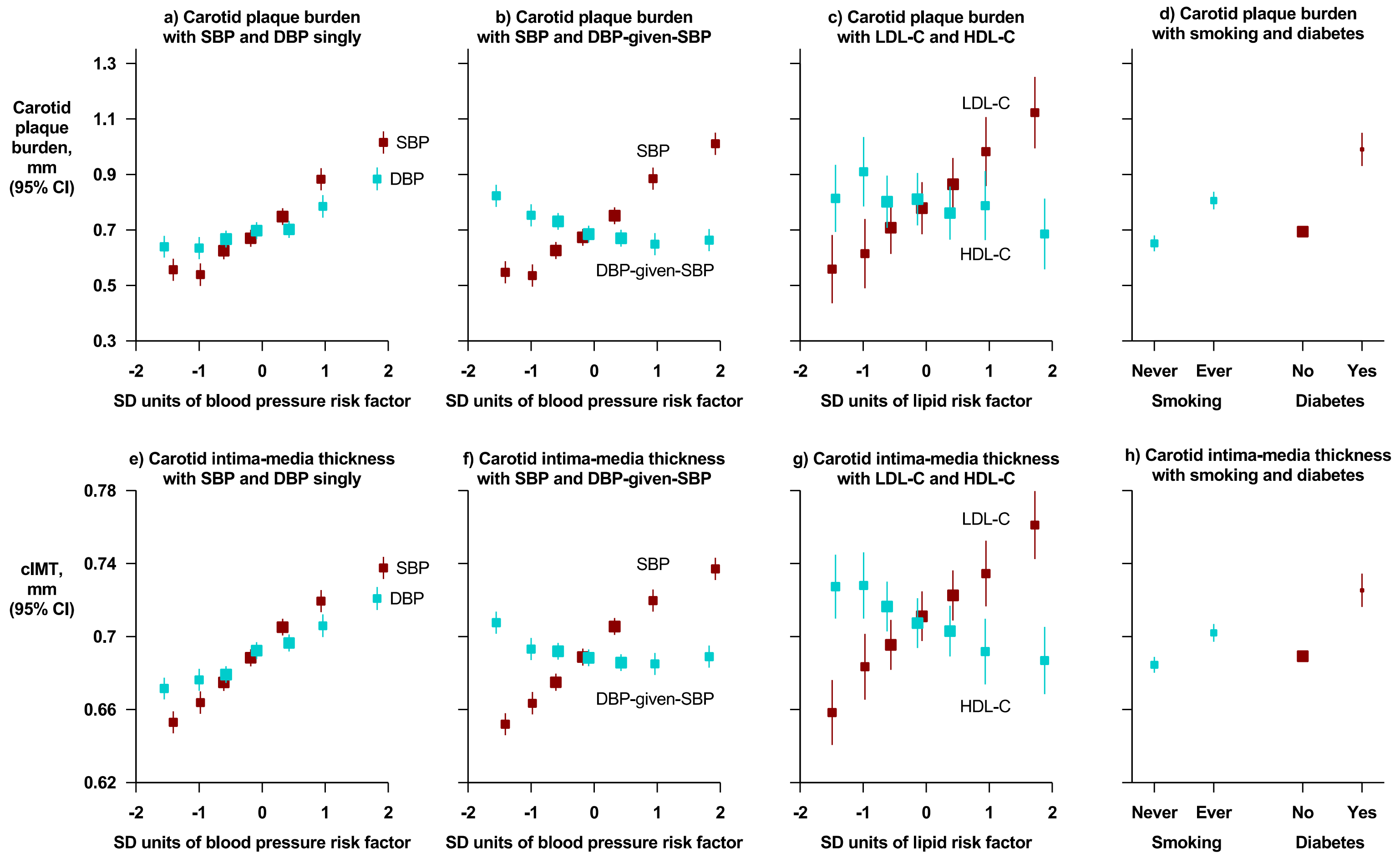


**eFigure 1: Associations of Major Cardiovascular Risk Factors at Baseline with Carotid Measures, in Participants Without a Stroke by Resurvey.** Blood pressure, smoking and diabetes associations in 23 021 participants; cholesterol associations in 2643 participants with measurements at baseline. Associations are adjusted for age, sex and area. SBP=Systolic blood pressure. DBP=Diastolic blood pressure. LDL-C=low-density lipoprotein cholesterol. HDL-C=high-density lipoprotein cholesterol. SD=standard deviation. SDs are: SBP 21 mmHg, DBP 11 mmHg, DBP-given-SBP 7 mmHg, LDL-C 26 mg/dL (0.7 mmol/L), HDL-C 11 mg/dL (0.3 mmol/L).

© 2019 Parish S et al. *JAMA Network Open.*

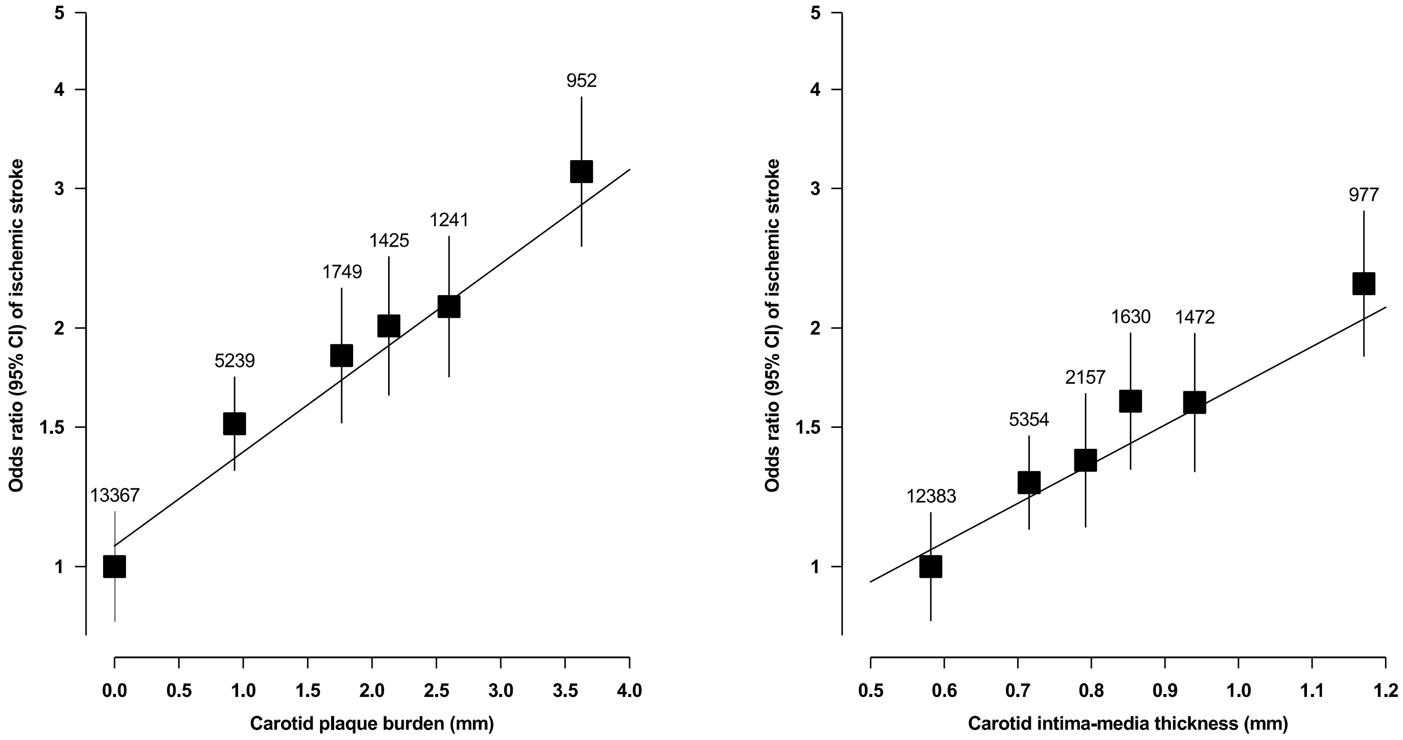


**eFigure 2: Association of Carotid Plaque Burden and Carotid Intima-media Thickness With Ischemic Stroke.** Based on 952 ischemic strokes in 23 973 participants without cardiovascular disease at baseline. Odds ratios are adjusted for age, sex and area. Participants were ranked by the respective carotid measure, and then groups allocated so that each group contained an equal number (~158) of strokes. The number of participants in each group is indicated.

© 2019 Parish S et al. *JAMA Network Open.*

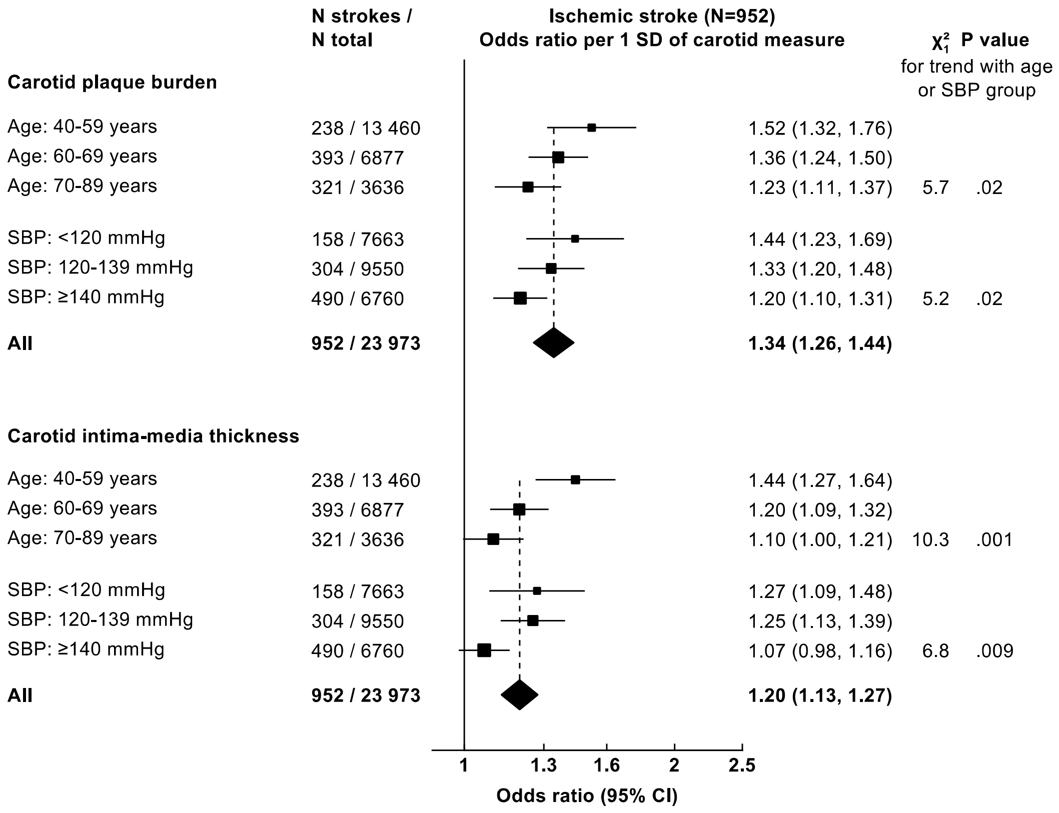


**eFigure 3: Associations of Carotid Measures With Ischemic Stroke, by Groups of Age at Resurvey and Baseline Systolic Blood Pressure (SBP).** Odds ratios are adjusted for age, sex and area. SD=standard deviation. SDs are: carotid plaque burden 1.1 mm, carotid intima-media thickness 0.16 mm.

© 2019 Parish S et al. *JAMA Network Open.*

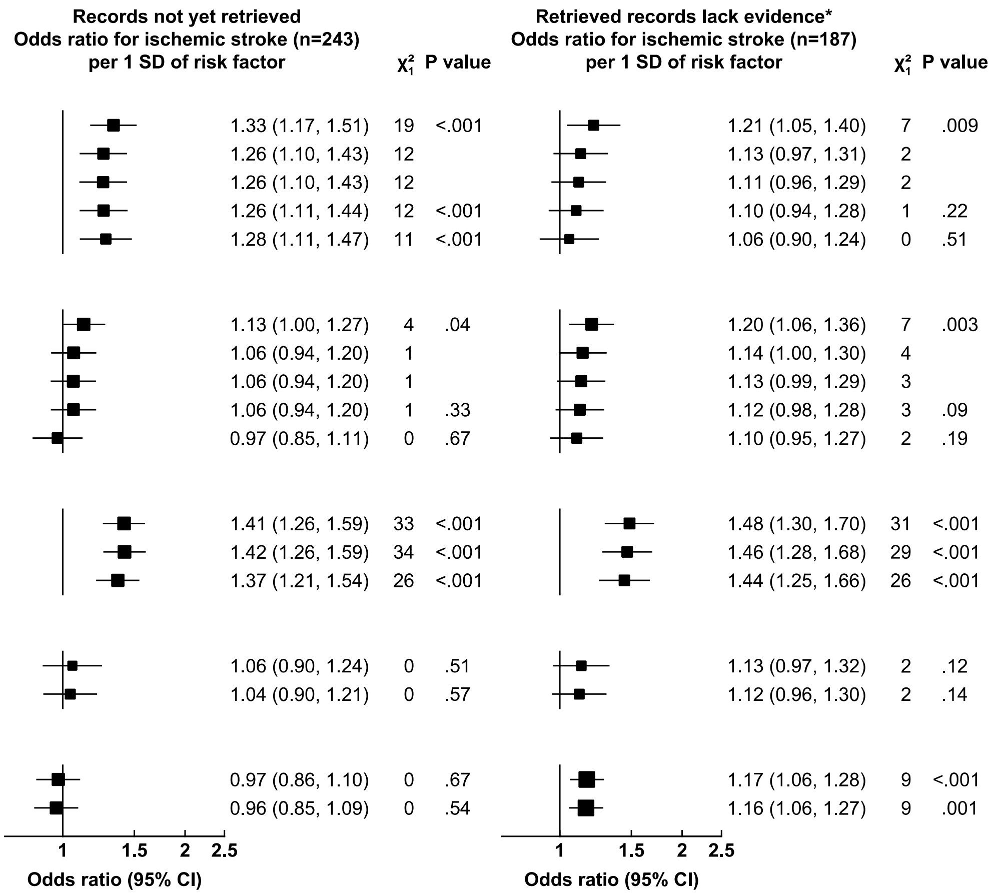

| | Records not yet retrieved: Odds ratio for ischemic stroke (n=243) per 1 SD of risk factor | $\chi_1^2$ | P value | Retrieved records lack evidence*: Odds ratio for ischemic stroke (n=187) per 1 SD of risk factor | $\chi_1^2$ | P value |
|---|---|---|---|---|---|---|
| **Carotid plaque burden** | | | | | | |
| Basic adjustment | 1.33 (1.17, 1.51) | 19 | <.001 | 1.21 (1.05, 1.40) | 7 | .009 |
| + blood pressure | 1.26 (1.10, 1.43) | 12 | | 1.13 (0.97, 1.31) | 2 | |
| + smoking | 1.26 (1.10, 1.43) | 12 | | 1.11 (0.96, 1.29) | 2 | |
| + diabetes | 1.26 (1.11, 1.44) | 12 | <.001 | 1.10 (0.94, 1.28) | 1 | .22 |
| + cIMT | 1.28 (1.11, 1.47) | 11 | <.001 | 1.06 (0.90, 1.24) | 0 | .51 |
| **cIMT** | | | | | | |
| Basic adjustment | 1.13 (1.00, 1.27) | 4 | .04 | 1.20 (1.06, 1.36) | 7 | .003 |
| + blood pressure | 1.06 (0.94, 1.20) | 1 | | 1.14 (1.00, 1.30) | 4 | |
| + smoking | 1.06 (0.94, 1.20) | 1 | | 1.13 (0.99, 1.29) | 3 | |
| + diabetes | 1.06 (0.94, 1.20) | 1 | .33 | 1.12 (0.98, 1.28) | 3 | .09 |
| + carotid plaque burden | 0.97 (0.85, 1.11) | 0 | .67 | 1.10 (0.95, 1.27) | 2 | .19 |
| **Systolic blood pressure** | | | | | | |
| Basic adjustment | 1.41 (1.26, 1.59) | 33 | <.001 | 1.48 (1.30, 1.70) | 31 | <.001 |
| + smoking + diabetes | 1.42 (1.26, 1.59) | 34 | <.001 | 1.46 (1.28, 1.68) | 29 | <.001 |
| + carotid plaque burden | 1.37 (1.21, 1.54) | 26 | <.001 | 1.44 (1.25, 1.66) | 26 | <.001 |
| **Smoking score** | | | | | | |
| Basic + BP + diabetes | 1.06 (0.90, 1.24) | 0 | .51 | 1.13 (0.97, 1.32) | 2 | .12 |
| + carotid plaque burden | 1.04 (0.90, 1.21) | 0 | .57 | 1.12 (0.96, 1.30) | 2 | .14 |
| **Diabetes** | | | | | | |
| Basic + BP + smoking | 0.97 (0.86, 1.10) | 0 | .67 | 1.17 (1.06, 1.28) | 9 | <.001 |
| + carotid plaque burden | 0.96 (0.85, 1.09) | 0 | .54 | 1.16 (1.06, 1.27) | 9 | .001 |



**eFigure 4: Associations of Carotid Measures and Major Cardiovascular Risk Factors With Unconfirmed Ischemic Strokes in 23 973 Participants.** Basic adjustment is age, sex and area. Smoking adjustment is for terms in the smoking score. SD=standard deviation. cIMT=carotid intima-media thickness. * Retrieved records for an index stroke lack evidence of ischemic stroke (see eMethods 2 in the Supplement).

© 2019 Parish S et al. *JAMA Network Open.*

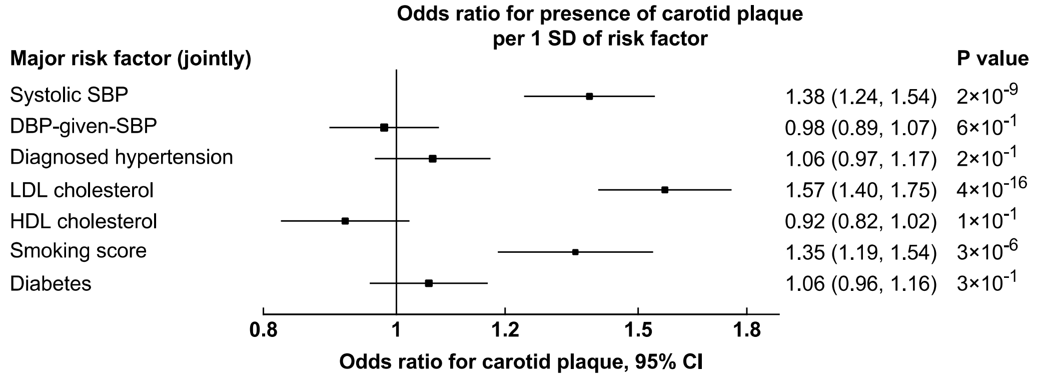


**eFigure 5: The Joint Effects of Major Cardiovascular Risk Factors Measured at Baseline on the Presence of Carotid Plaque (>1.5 mm) in 2643 Participants Without Prior Cardiovascular Disease By Resurvey.** Associations are adjusted for age, sex and area. The smoking score (scaled to have an SD of 1) includes the predictor terms for smoking status (never, occasional, ex-regular or current regular smoker) and the number of cigarettes smoked. In the fitted model, the smoking score is 0 for never smokers, 0.31 for an occasional smoker, -0.23 for an ex-smoker and 1.32 plus 0.04 per cigarette per day for current smokers.Thus, a current smoker of 15 cigarettes per day would have a score of about 1. For consistency with other factors the diabetes and diagnosed hypertension effects are displayed as the effect per SD of the condition prevalence; to convert to the effect with having the condition, divide the values in the figure by the SD of the respective prevalence. SDs are: diagnosed hypertension prevalence 0.29, diabetes prevalence 0.20, carotid plaque burden 1.1 mm, cIMT 0.16 mm; other SDs as in eFigure 1. BP=blood pressure. DBP-given-SBP=diastolic BP given systolic BP. LDL=low density lipoprotein. HDL=high density lipoprotein. SD=standard deviation.

© 2019 Parish, S, et al. *JAMA Network Open.*

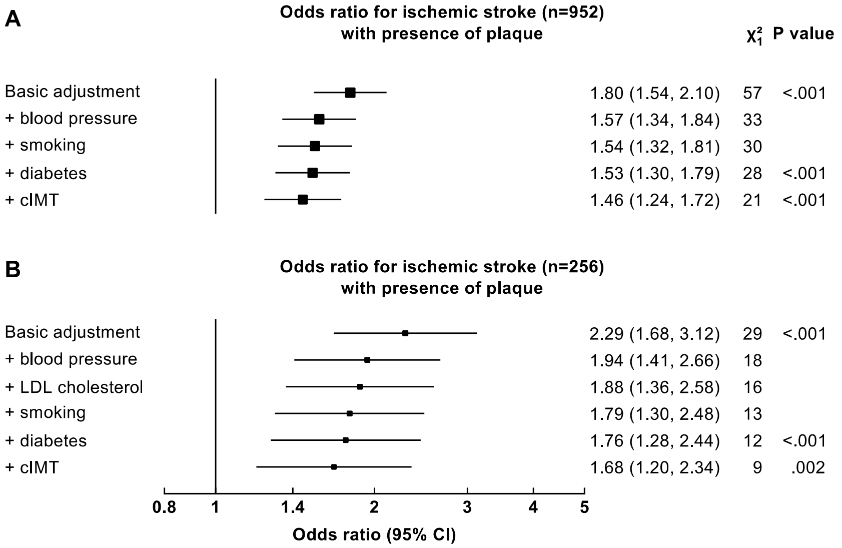


**eFigure 6: Association of Presence of Carotid Plaque (>1.5 mm) With Ischemic Stroke: A in All 23 973 Participants, B in 2899 Participants With Lipid Measurements.** Basic adjustment is age, sex and area. LDL=low density lipoprotein. cIMT=carotid intima-media thickness

© 2019 Parish, S, et al. *JAMA Network Open.*
